# New Players in the Same Old Game: Disturbance of Group 2 Innate Lymphoid Cells in HIV-1 and *Mycobacterium leprae* Co-infected Patients

**DOI:** 10.1371/journal.pntd.0004030

**Published:** 2015-09-03

**Authors:** Pedro Henrique Papotto, Solange Maeda, Jane Tomimori, Marília Brasil Xavier, Luiz Vicente Rizzo, Esper Georges Kallas, Karina Inácio Carvalho

**Affiliations:** 1 Hospital Israelita Albert Einstein, Instituto Israelita de Ensino e Pesquisa, São Paulo, Brazil; 2 Universidade Federal de São Paulo, Dermatology Department, São Paulo, Brazil; 3 Universidade Federal do Pará, Dermatology Department, Pará, Brazil; 4 Universidade de São Paulo, Faculdade de Medicina, Disciplina de Imunologia Clínica e Alergia (LIM60), São Paulo, Brazil; University of Tennessee, UNITED STATES

## Abstract

Leprosy control is achieved through a fine-tuning of T_H_1 and T_H_2 immune response pattern balance. Given the increasing epidemiological overlay of HIV and *M*. *leprae* infections, immune response in co-infected patients consists in an important contemporary issue. Here we describe for the first time the innate lymphoid cells compartment in peripheral blood of leprosy and HIV/*M*. *leprae* co-infected patients, and show that co-infection increases group 2 innate lymphoid whilst decreasing group 1 innate lymphoid cells frequencies and function.

## Author Summary

*Mycobacterium leprae* is a clinical relevant pathogen that can lead to leprosy upon infection. This chronic infectious disease is characterized by the appearance of skin and peripheral nerve lesions. Normally, a healthy immune system is able to control the infection and impede the generation of lesions. However, immunosuppressive conditions may reduce the fitness of host’s immune system, with consequences of disease progression and pathogen spreading. HIV is the major cause of pathogen-associated immunodeficiency, and during the past decades, an epidemiological overlay of *M*. *leprae* and HIV infections spectra has been observed. Thus, the increase of co-infection cases raised the interest of the scientific community in this subject. Accordingly, our group has previously shown that co-infected patients presented a shift in the type of cellular immune response. Nevertheless, in the past few years a new group of innate lymphoid cells (ILCs) was shown to play an important role in the initiation and maintenance of effective immune responses. Here we describe for the first time the circulating ILCs subsets in leprosy, HIV and *M*. *leprae*/HIV co-infected patients. Additionally, we found that the co-infection scenario leads to a shift in different ILCs population, concordant with those observed previously for T lymphocytes and innate immunity cells.

## Introduction

Leprosy is a chronic infectious disease caused by the intracellular bacillus *Mycobacterium leprae*. Although usually asymptomatic, some subjects develop the clinical form of disease, exhibiting skin and peripheral nerves lesions [1]. In 2013, the Brazilian Healthy Department, registered 31.988 new cases of leprosy, with 84% of cure [2]. However, Menezes et al., describe an increasing number of HIV-1/*M*. *leprae* co-infected patient admitted in a center of leprosy treatment in Rio de Janeiro state [3]. In a referral HIV and leprosy center in Amazonas state, Talhari et al., observed in 13-year follow-up study higher leprosy prevalence among HIV-positive individuals [4].

Normally, clinical forms of the disease can be divided under the light of T_H_1/T_H_2 paradigm; being tuberculoid, or paucibacillary, leprosy linked to a T_H_1 response pattern, and lepromatous, or multibacillary, leprosy linked to a T_H_2 response profile. Lately, T_H_17 cells were shown to play a role in disease pathogenesis, especially in lesion formation and *M*. *leprae* control [5]. Moreover, it was suggested that T_H_17 cells contribute to pathogen clearance, when T_H_1 or T_H_2 polarization is ineffective [6].

As in other chronic and latent infections, the control of pathogen load is highly dependent on the immunological fitness of the host. Therefore, immunosuppressive factors contribute to disruption of host-pathogen homeostasis, and may account to aggravation of disease and enhancement of *M*. *leprae* transmission [7]. HIV-1 infection is the most common acquired immunodeficiency. In HIV-1/*Mycobacterium tuberculosis* co-infection, the suppression of immune system caused by HIV was shown to accelerate disease progression [8]. Likewise, in HIV-1/*M*. *leprae* co-infected patients our group observed an imbalance towards T_H_2 responses, with lower CD4:CD8 ratios and higher IL-4 levels when compared to healthy controls [9]. Furthermore, patients co-infected with HIV-1 and *M*. *leprae* exhibited lower frequency of plasmacytoid dendritic cells (pDCs) and Natural Killer T (NKT) cells when compared to healthy controls [9,10], indicating that innate immunity cells are also affected during co-infection.

In recent years, innate lymphoid cells (ILCs) have emerged as important effector cells of innate immunity, responsible for bridging innate and adaptive responses. Recently, ILCs have been divided in three major groups, regarding their effector function and cytokine production profile [11]. These groups correlate well with T helper lymphocyte subsets. Hence, Group 1 ILCs (here referred to as ILC1) are characterized by the production of “T_H_1” cytokines, such as IFN-γ and TNF-α. Natural Killer (NK) cells are comprised in this group, and the ontogeny of other IFN-γ-producing ILCs is still controversial [11,12]. Group 2 ILCs (ILC2) is an important innate source of “T_H_2” cytokines, such as IL-4, IL-5 and IL-13. These cells respond to IL-25 and IL-33 and are associated with protection in helminthes infection and allergic asthma [11,12]. Finally, Group 3 ILCs (here referred to as ILC3) is composed of the well known Lymphoid-tissue inducer (LTi) cells and NCR^+^ and colitogenic NCR^-^ non-LTi cells. These cells produce IL-17A and/or IL-22 and are involved in different bacterial infections [11,12].

There are only a few studies regarding the effects of HIV or SIV infection on ILCs subsets. In two macaque studies, SIV infection led to a disturbance of IL-17-producing ILC3 cells [13,14]. However, in HIV-infected patients IL-22-producing ILC3 cells were still fully functional [15]. Interestingly, there are no studies on ILCs in leprosy and, consequently, in HIV/*M*. *leprae* co-infection. Thus, in the present study we evaluated the frequency and function of all three ILCs subsets in the peripheral blood of *M*. *leprae* and HIV mono-infected patients and HIV/*M*. *leprae* co-infected patients.

## Methods

### Ethics Statement

Volunteers were recruited at the Federal University of São Paulo and the Federal University of Pará, Brazil. Written informed consent, approved by the Institutional Review Board, (Comitê de Ética em Pesquisa Humana da Universidade Federal de São Paulo/UNIFESP and Comitê de Ética da Universidade Federal do Pará) were obtained from all volunteers, according to the Brazilian Ministry of Health Guidelines and the Declaration of Helsinki.

### Subjects

Leprosy patients were treated according to World Health Organization Guidelines [16], and co-infected patients were treated with the appropriate multidrug therapy (MDT) for paucibacillary (PB) and multibacillary (MB) leprosy. The initial treatment for patients with HIV mono- and co-infection o was defined using modified criteria adopted by the Brazilian Ministry of Health at the time of sample collection that includes patients with a CD4^+^ T cell count of < 350 cells/μL or any AIDS-defining clinical condition [17]. These guidelines have been recently updated [18]. The HIV mono-infected and co-infected patients received highly active antiretroviral therapy (HAART) and multidrug therapy (MDT). Patients with immune reconstitution inflammatory syndrome, with leprosy reactions and under systemic corticosteroid and/or thalidomide were not included in the present study, to avoid potential interference in the immune parameters as described in a previous studies. The study subjects were divided into four groups: 16 healthy controls (Healthy) and 11 HIV seropositive patients (HIV), most of whom had CD4+ T cell counts of less than 400 cells/μL, 7 patients infected with *M*. *leprae* (Leprosy), and 10 co-infected patients with *M*. *leprae* and HIV co-infection (Dual), recruited at Leprosy Outpatient Clinics at both sites. In this the major presentations of leprosy were multibacillary form rather than paucibacillary form. For all analysis we grouped the leprosy mono-infected and co-infected patients (*leprosy per se)* (S1 Table).

### Flow Cytometry

To characterize and define ILCs subsets immune staining of surface molecules was performed using anti-Lin (CD11c, CD16, CD3 and CD19), CD127 (clones: HIL-7R-M21 and R34.34), CD25 (clone: M-A251), CD45 (clone: 2D1), CRTH2 (clone: BM16), CD161 (clone: DX12), NKp44 (clone: Z231), CD56 (clone: B159) antibodies by flow cytometry. The amine Aqua dye (Invitrogen, Carlsbad, CA, USA) was used to exclude dead cells in all samples. Briefly, peripheral blood mononuclear cells (PBMC) were collected from all subjects and frozen in liquid nitrogen until usage. After thawing, cells were stained with aforementioned antibodies and acquired on a LSR Fortessa flow cytometer (BD Biosciences) [9]. For the cytokine production measurement, PBMC were thawed and incubated in the presence of 100 ng/ml phorbol 12-myristate 13-acetate (PMA–Sigma) and 500 ng/mL ionomycin (Sigma). After 1 h at 37°C and 5%CO_2_, brefeldin A (5mg/ml) was added (BD Biosciences). After incubation for 16 h, cells were washed, and incubated with monoclonal antibodies for surface. Cells were washed and fixed/permeabilized using fixation/permeabilization reagents from Life Technologies in accordance with manufacturer’s instructions. Cells were then washed and incubated with anti-IL-4 (clone: MP4-25D2), IL-13 (clone: JES10-5A2), TNF-α (clone: MAb11), and IL-17 (clone: SCPL1362) and acquired in a LSR Fortessa flow cytometer (BD Biosciences). Fluorescence minus one (FMO) was used as gating strategy for surface panels. For intracellular analysis, was used the unstimulated cells for each panels. All samples were acquired using FACSDiva software (BD Biosciences), and then analyzed with FlowJo software version 9.7.8 (Tree Star). Fluorescence voltages were determined using matched unstained cells. Compensation was carried out using CompBeads (BD Biosciences) single stained with all fluorochromes used in the experiments. Samples were acquired to reach at least 1,000,000 events.

Groups were compared using non-parametric models; data were reported with median and 25–75% interquartile range. *p* values were considered significant if below 0.05. Results are expressed in medians and interquartile ranges (IQR).

## Results

Study subjects were divided into four distinct groups, 16 healthy controls (Healthy), 11 HIV-infected patients (HIV), 7 patients infected with *M*. *leprae* (Leprosy) and 10 co-infected patients with *M*. *leprae* and HIV co-infection (Dual). Demographic details can be found at S1 Table. The median age of all participants was 36 years and no difference in age distribution was found between groups. Most of the subjects were male (70.21%). All patients were properly treated with HAART and/or MDT therapy.

**Fig 1:**
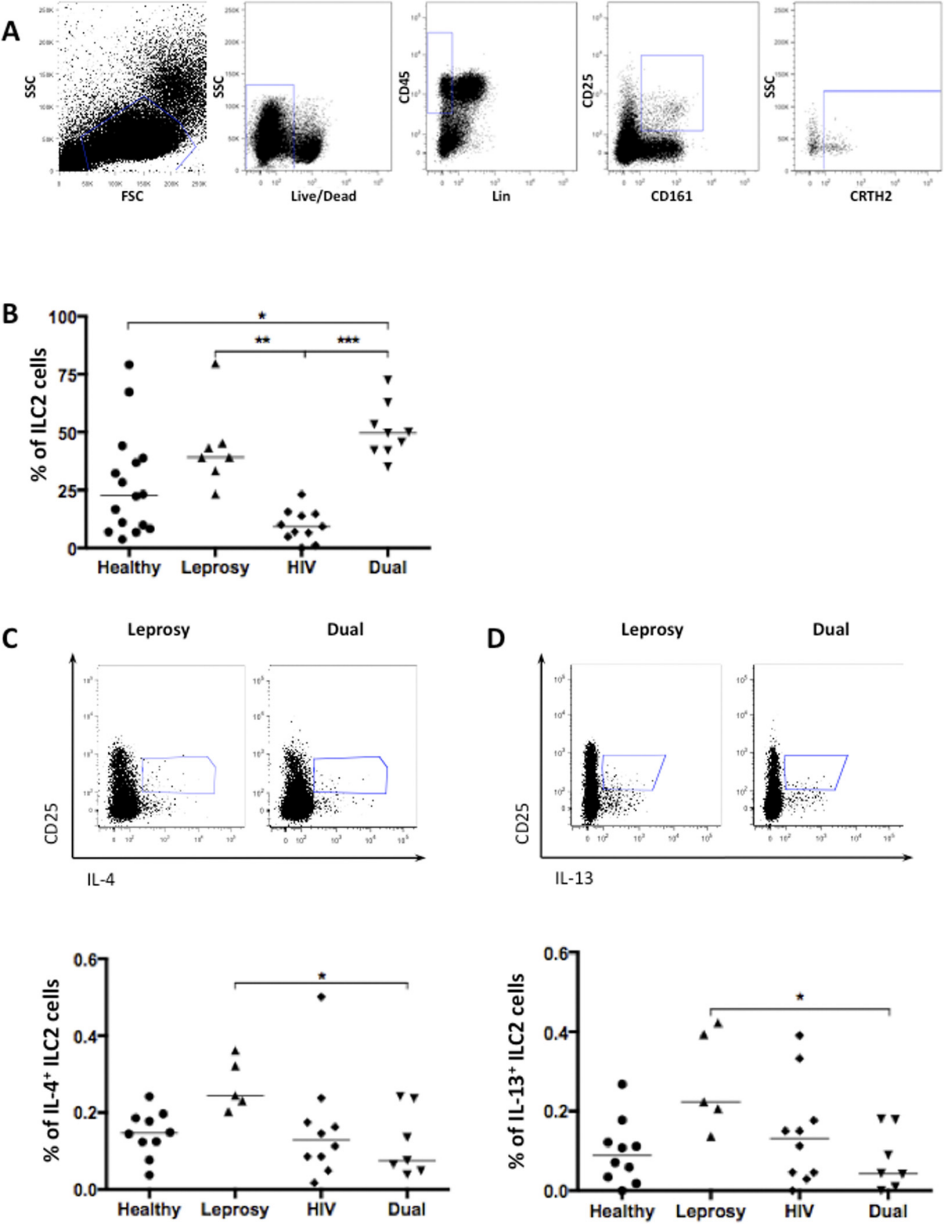
*M*. *Leprae*/HIV-1 co-infected patients present a disturbance of circulating ILC2 cells. *A*, gate strategy used to define group 2 innate lymphoid cells based on the expression of surface proteins. *B*, frequencies of circulating ILC2 cells defined as Lin^-^CD45^+^CD161^+^CD25^+^CRTH2^+^ from healthy (n = 16), M. leprae-infected (n = 7), HIV-1-infected (n = 11) and co-infected (n = 10) cohorts. *C*, intracellular staining of IL-4 on Lin^-^CD45^+^CD25^+^ cells. Frequencies of IL-4 producing cells within healthy (n = 10), *M*. *leprae*-infected (n = 5), HIV-1-infected (n = 10) and co-infected (n = 7) groups. *D*, intracellular staining of IL-13 on Lin^-^CD45^+^CD25^+^ cells. Frequencies of IL-13 producing cells within healthy (n = 10), M. leprae-infected (n = 5), HIV-1-infected (n = 10) and co-infected (n = 7) groups. Each dot represents an individual, and bars indicate medians in the graphs. Statistical analysis was performed using the Kruskal-Wallis test. * p<0.05; ** p<0.001; ***p<0.0001.

Group 2 innate lymphoid cells were defined as Lin^-^CD45^+^CD161^+^CD25^+^CRTH2^+^ cells (Fig 1A). Consistent with previous data from our group, HIV-1/*M*. *leprae* co-infection generates a T_H_2-polarized environment [9], here demonstrated by an increase in ILC2 cells frequencies when compared co-infected HIV-1/*M*. *leprae* patients (dual) with HIV-1 mono-infected patients and healthy controls (50.00, IQR 42.1–67.30 vs. 9.30, IQR 4.80–14.70, p<0.001, and 50.00, IQR 42.1–67.30 vs. 22.40, IQR 8.20–33.80, p<0.01 respectively) (Fig 1B). Additionally, *M*. *leprae* mono-infected patients exhibited higher frequencies of ILC2 cells when compared to HIV-1 mono-infected patients (39.10, IQR 33.30–45.30 vs. 9.33, IQR 4.88–14.70; p<0.01) (Fig 1B). When compared to healthy controls, *M*. *leprae* mono-infected patients showed a trend, although not significant, increase in the frequency of ILC2 cells (39.10, IQR 33.30–45.30 vs. 22.75, IQR 9.07–37.80, p>0.05, respectively). Altogether, these results indicate that in HIV-1/*M*. *leprae* co-infected patients it is observed an higher frequencies of ILC2 compartment when compared to healthy controls and HIV-1 mono-infected patients.

ILC2 cells have been shown to secrete different T_H_2 cytokines. Here we analyzed the production of both IL-4 and IL-13 by Lin^-^CD45^+^CD25^+^ cells. As expected, these cells produced both IL-4 (Fig 1C) and IL-13 (Fig 1D), consistent with ILC2 phenotype. Surprisingly, ILC2 cells from HIV-1/*M*. *leprae* co-infected patients exhibited lower frequencies of IL-4- (Fig 1C) and IL-13-producing cells (Fig 1D) when compared to cells from *M*. *leprae* mono-infected patients (0.245, IQR 0.217–0.342 vs. 0.075, IQR 0.048–0.237; p<0.05 and 0.224, IQR 0.172–0.408 vs. 0.043, IQR 0.009–0.179; p<0.05, respectively). These findings demonstrate that HIV-1/*M*. *leprae* co-infection although promoting an increase in ILC2 cells frequency does not increase their functional capacity when compared to other groups. Moreover co-infection scenario impairs ILC2 ability to produce T_H_2 cytokines, in comparison to *M*. *leprae* mono-infected patients.

**Fig 2:**
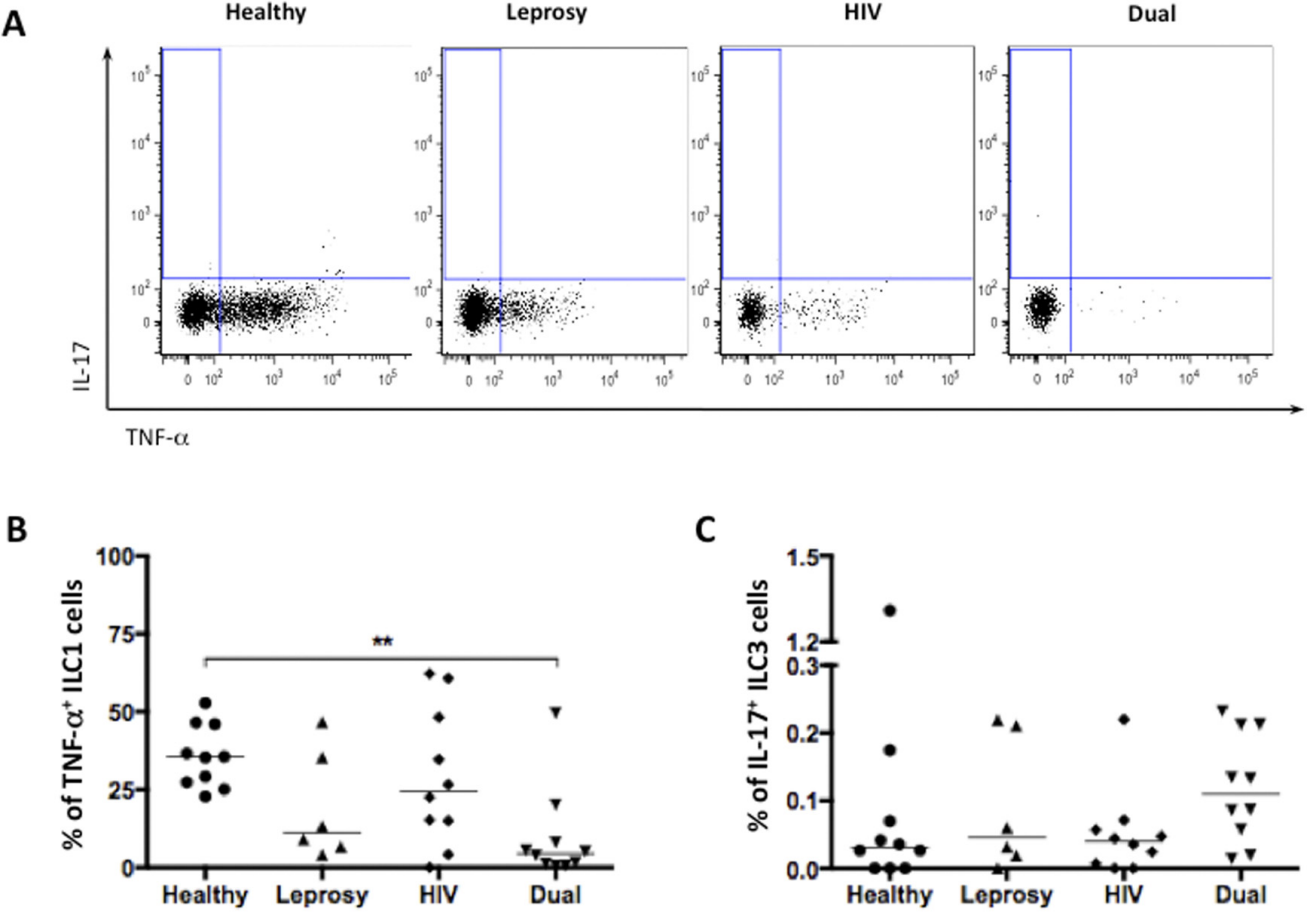
*M*. *Leprae*/HIV-1 co-infection led to a decrease of circulating ILC1 cells. *A*, gate strategy used to define group 1 and group 3 innate lymphoid cells based on the production of TNF-α and IL-17 by Lin^-^CD45^+^CD56^+^ cells. *B*, Frequencies of TNF-α-producing cells, defined as ILC1 cells, from healthy (n = 10), *M*. *leprae*-infected (n = 6), HIV-1-infected (n = 10) and co-infected (n = 10) patients. *C*, Frequencies of IL-17-producing cells, defined as ILC3 cells, from healthy (n = 10), *M*. *leprae*-infected (n = 6), HIV-1-infected (n = 10) and co-infected (n = 10) patients. Each dot represents an individual, and bars indicate medians in the graphs. Statistical analysis was performed using the Kruskal-Wallis test. * p<0.05; ** p<0.001; ***p<0.0001.

Additionally to ILC2 subset, we evaluated the frequencies of ILC1 and ILC3 populations on the peripheral blood of all subjects. ILC1 cells were defined as Lin^-^CD45^+^CD56^+^TNF-α^+^ cells and ILC3 subset was defined as Lin^-^CD45^+^CD56^+^IL-17^+^ cells (S1 Fig and Fig 2A). Conversely to the increase of ILC2 cells frequencies in HIV/*M*. *leprae* co-infected patients, we found a decrease in ILC1 cells frequencies when compared to healthy controls (Fig 2B) (4.52, IQR 0.89–14.08 vs. 35.50, IQR 26.25–46.25; p<0.01). No differences were found in ILC3 cells frequencies between groups (Fig 2C).

## Discussion

To our knowledge, this is the first study to investigate and describe ILCs subsets in leprosy and HIV-1/*M*. *leprae* co-infected patients. Moreover, this is the first time that a complete profile of the three different ILCs subsets was carried out in HIV-1 infected patients, as the only study regarding this question focused on ILC3 cells [15]. One limitation of this study, however, is the relatively small sample size, which may have affected the ability to identify more subtle changes between groups. Nevertheless, we were able to identify major changes in ILCs compartment caused by HIV-1/*M*. *leprae* co-infection. To define the three major groups of ILCs we chose to use a functional criterion (i.e. cytokine secretion) in order to avoid misconception of ILCs subpopulations [12]. Consequently, when using the ILC1 term we are referring to both NK cells and T_H_1 (i.e., TNF-α) cytokines-producing ILCs [11]. Similarly, here we used the ILC3 term to comprise LTi cells, NCR^+^ and NCR^-^ cells, based on their ability to produce IL-17 [11]. Finally, ILC2 cells were defined both by their IL-4 and IL-13 secretory capacities and surface molecules, because this subset is composed by only one known population of cells [11].

Here we found that patients with HIV-1 and *M*. *leprae* co-infection showed an increase in ILC2 cells and a decrease in ILC1 cells frequencies in peripheral blood, when compared to healthy subjects. This data is in line with previous findings from our group, which showed a skewing in cellular immune response towards a T_H_2 bias [9]. Thus, our data add new evidences to the hypothesis that HIV-1 in patients with an ongoing infection with *M*. *leprae* promotes a disturbance in T_H_1 and T_H_2 balance, sustaining a T_H_2 environment. Even though not conclusive, our results indicate that HIV-1 infection is responsible for enhancing this T_H_2 shift. However, it is not clear if this skewing in immune response is a viral escape mechanism or an attempt from the immune system to control the virus. Although higher in frequencies, ILC2 cells from HIV-1/*M*. *leprae* co-infected patients produce less IL-4 and IL-13, when compared to *M*. *leprae* mono-infected patients. Additionally, circulating ILC2 cells in HIV-1 mono-infected patients are present in similar frequencies and with comparable functional capacity of healthy subjects, suggesting that HIV-1 is not responsible for inducing a relevant T_H_2 response, *per se*. Therefore, suggesting that HIV-1 infection impairs T_H_2 response, through decrease of functional response, and the resulting increase in ILC2 frequencies is an attempt from the immune system to rescue this phenotype. Concurrently with the increase of circulating ILC2 frequencies it was observed a decrease of circulating ILC1 frequencies in co-infected patients when compared to their healthy counterparts, further supporting this notion of an unbalancing of T_H_1/T_H_2 responses. Of note, to better understand the immunological landscape in HIV-1/*M*. *leprae* co-infection it would be crucial to dissect the immune response of ILCs and other immune cells located in the site of *M*. *leprae* infection (i.e. in the skin and nerves).

We found no differences in the frequencies of ILC3 subset from HIV-1/*M*. *leprae* co-infected patients when compared to healthy subjects and mono-infected patients, as opposed to previous findings that showed IL-17-producing ILCs cells depletion in SIV infected macaques [14]. Nonetheless, in the study led by Xu, cells were obtained from mucosal tissues. Moreover, these findings were obtained in an experimental macaque model [14]. Conversely, Fernandes and colleagues looked on IL-22-producing ILC3 cells population on mucosal surfaces of HIV-1 infected individual and found no differences in their frequencies when compared to healthy controls [15]. Although functionally different ILC3 subsets, the conditions in this study are the most comparable to ours, adding strength to our findings that HIV-1 does not alter ILC3 cells population.

Finally, in this study we added new information to the growing body of data that shows that ILCs have effector functions that parallels T helper cells subsets during infection. Additionally, our main finding that HIV-1 infection has a major effect on ILC2 cells demonstrate that these cells might have a role during viral infections, and not only on asthma and helminthes infections [11].

## Supporting Information

S1 Fig Gate strategy used to define cytokine-producing ILCs subpopulations. *A*, intracellular staining of IL-13 and IL-4 on Lin^-^CD45^+^CD25^+^ cells. *B*, strategy used to define group 1 and group 3 innate lymphoid cells based on the production of TNF-α and IL-17 by Lin^-^CD45^+^CD56^+^ cells.
(TIFF)

S1 Table Demographic and clinical characteristics of participants. (DOCX)
