# Supplementary figures and images for: New Players in the Same Old Game: Disturbance of Group 2 Innate Lymphoid Cells in HIV-1 and Mycobacterium leprae Co-infected Patients
Source: PLoS Negl Trop Dis. 2015 Sep 3;9(9):e0004030. doi: 10.1371/journal.pntd.0004030 (PMC4559394; doi:10.1371/journal.pntd.0004030)

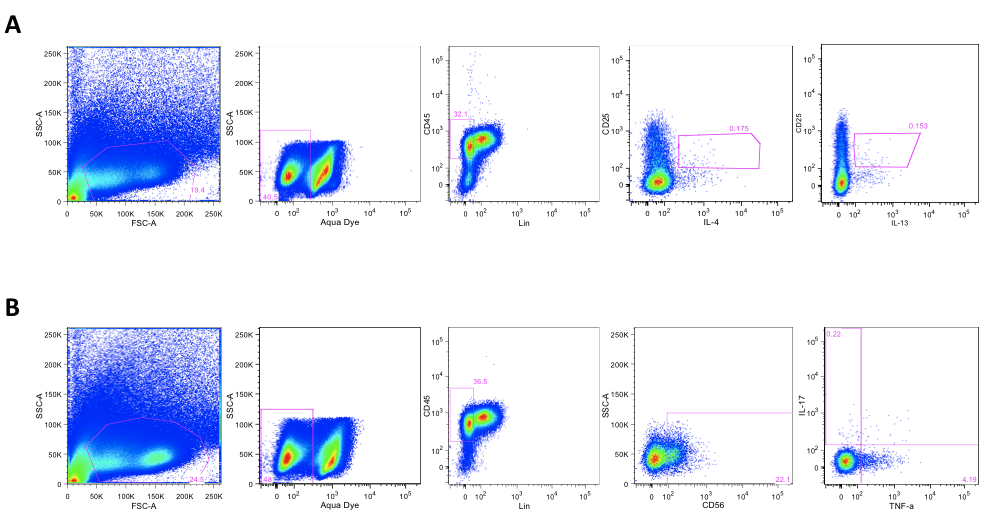

Supplement: S1 Fig — A, intracellular staining of IL-13 and IL-4 on Lin-CD45+CD25+ cells. B, strategy used to define group 1 and group 3 innate lymphoid cells based on the production of TNF-α and IL-17 by Lin-CD45+CD56+ cells. (TIFF) [file pntd.0004030.s001.tiff]
