# Supplementary material for: New Players in the Same Old Game: Disturbance of Group 2 Innate Lymphoid Cells in HIV-1 and Mycobacterium leprae Co-infected Patients
Source: PLoS Negl Trop Dis. 2015 Sep 3;9(9):e0004030. doi: 10.1371/journal.pntd.0004030 (PMC4559394; doi:10.1371/journal.pntd.0004030)
Supplement: S1 Table — (DOCX) [file pntd.0004030.s002.docx]

**Table 1:** Demographic and clinical characteristics of participants.

| **Case Number** | **Group** | **Age** | **Gender** | **Leprosy Classification** |
| --- | --- | --- | --- | --- |
| CN01 | Healthy | 28 | Female | ------ |
| CN03 | Healthy | 35 | Female | ------ |
| CN07 | Healthy | 38 | Female | ------ |
| CN08 | Healthy | 23 | Female | ------ |
| CN09 | Healthy | 45 | Female | ------ |
| CN10 | Healthy | 29 | Female | ------ |
| CN12 | Healthy | 30 | Female | ------ |
| CN13 | Healthy | 35 | Male | ------ |
| CN116 | Healthy | 40 | Male | ------ |
| CN129 | Healthy | 21 | Male | ------ |
| PCN03 | Healthy | 22 | Female | ------ |
| PCN08 | Healthy | 38 | Male | ------ |
| M001 | Healthy | 36 | Female | ------ |
| HB128 | Healthy | 35 | Male | ------ |
| HB08 | Healthy | 37 | Male | ------ |
| HB09 | Healthy | 35 | Male | ------ |
| H10 | Leprosy | 37 | Female | Multibacillary |
| L03 | Leprosy | 40 | Male | Multibacillary |
| L05 | Leprosy | 42 | Female | Multibacillary |
| L06 | Leprosy | 50 | Male | Multibacillary |
| PH04 | Leprosy | 64 | Male | Multibacillary |
| PH07 | Leprosy | 49 | Male | Multibacillary |
| PH03 | Leprosy | 37 | Male | Paucibacillary |
| 1021 | HIV | 50 | Male | ------ |
| 1033 | HIV | 45 | Male | ------ |
| 1054 | HIV | 41 | Male | ------ |
| 1080 | HIV | 21 | Male | ------ |
| 1097 | HIV | 39 | Male | ------ |
| 1142 | HIV | 43 | Male | ------ |
| 2011 | HIV | 34 | Male | ------ |
| 2016 | HIV | 28 | Male | ------ |
| 2033 | HIV | 40 | Male | ------ |
| 2039 | HIV | 32 | Male | ------ |
| 2042 | HIV | 42 | Male | ------ |
| H/L06 | Dual | 43 | Female | Multibacillary |
| H/L01 | Dual | 53 | Male | Paucibacillary |
| H/L03 | Dual | 30 | Female | Paucibacillary |
| HH07 | Dual | 40 | Male | Multibacillary |
| HH08 | Dual | 45 | Female | Multibacillary |
| HH09 | Dual | 34 | Male | Multibacillary |
| HH10 | Dual | 25 | Male | Paucibacillary |
| P2HH | Dual | 32 | Male | Paucibacillary |
| PHH15 | Dual | 28 | Male | Paucibacillary |
| PHH13 | Dual | 34 | Male | Paucibacillary |
